# Breast Cancer Vlogs on YouTube: Descriptive and Content Analyses

**DOI:** 10.2196/66812

**Published:** 2025-03-31

**Authors:** Nina Morena, Elly Dimya Htite, Yitzchok Ahisar, Victoria Hayman, Carrie A Rentschler, Ari N Meguerditchian

**Affiliations:** 1 Department of Art History and Communication Studies, McGill University, McCall McBain Arts Building, Room 155, 853 Sherbrooke Street West, Montreal, QC, H3A 0G5, Canada, 1 514-398-2850; 2 Faculty of Medicine, McGill University, Montreal, QC, Canada; 3 Department of Surgery, General Surgery, University of British Columbia, Vancouver, BC, Canada; 4 Department of Surgery, Faculty of Medicine, McGill University, Montreal, QC, Canada; 5 St Mary's Research Centre, Montreal, QC, Canada; 6 Gerald Bronfman Department of Oncology, Faculty of Medicine, McGill University, Montreal, QC, Canada

**Keywords:** breast cancer vlog, YouTube, social media, experience, video, content analysis, breast, cancer, women, oncology, descriptive analysis

## Abstract

**Background:**

Many women with breast cancer document their experiences in YouTube vlogs, which may serve as peer-to-peer and community support.

**Objective:**

This study aimed to determine (1) the forms of content about breast cancer that tend to be discussed in vlogs, (2) the reasons why women choose to vlog their breast cancer experiences, and (3) the potential for breast cancer vlogs to serve as an alternative or complement to peer-to-peer support as well as a site of digital community overall.

**Methods:**

YouTube was searched in incognito mode in November 2023 using the search terms “breast cancer vlog.” A maximum of 10 videos/creator were included based on viewership and date created. Video characteristics collected included title; length; number of views, likes, comments; and playlist inclusion. Videos were assessed for sponsorship; presence of explanation and discussion on breast cancer; type of content; and themes. Creator characteristics included age, location, and engagement approaches. Descriptive and content analyses were performed to analyze video content and potential areas where peer-to-peer support may be provided.

**Results:**

Ninety vlogs by 13 creators were included, all from personal accounts. The mean (SD) video length, number of views, and number of comments were 21.4 (9.1) minutes, 266,780 (534,465), and 1485 (3422), respectively. Of the 90 videos, 35 (39%) included hashtags, and 11 (12%) included paid sponsorships. The most common filming location was the home (87/90; 97%), followed by the hospital (28/90; 31%) and car (19/90; 21%). Home vlogs were most often set in the living room (43/90; 44%), bedroom (32/90; 33%), or kitchen (20/90; 21%). Thirty-four of 60 videos (57%) included treatment visuals and physical findings. Creators addressed motivation for vlogging in 44/90 videos (49%); the two most common reasons were wanting to build a community and helping others. In 42/90 videos (47%), creators explicitly expressed emotion. Most common themes were treatment (77/90; 86%), mental health (73/90; 81%), adverse effects (65/90; 72%), appearance (57/90; 63%), and family relationships (33/90; 37%). Patient-directed advice was offered in 52/90 videos (58%), mostly on treatment-related issues. In 51/90 videos (57%), creators provided explicit treatment definitions. Chemotherapy was discussed in 63/90 videos (70%); surgery in 52/90 (58%), primarily mastectomy; radiation in 27/90 (30%); and general adverse effects in 64/90 (71%). Twenty-two of 90 videos (24%) were about a new diagnosis. When mentioned (40/90; 44%), the most common creator location was the United States. When mentioned (27/90; 30%), the most common age was 20‐29 years.

**Conclusions:**

The dedication to building community support by vlog creators, and the personal nature of their storytelling, may make vlogs a potential resource for peer-to-peer support.

## Introduction

Vlogs, or “video blogs,” are personally and individually created experiential videos based on wide-ranging topics, usually posted to YouTube. A popular subset of vlogs are created by women with breast cancer, in which they document their breast cancer experiences in a publicly accessible digital format. As videos that tend to invite viewers into the lives of their creators, vlogs typically receive high levels of engagement and draw audiences who will continue watching in order to keep up with what creators are doing next. Online tools such as these vlogs are important parts of patients’ experience with processing and managing chronic illness [1]. They can help patients find support, community, and information in spaces they may not have access to in real life [2]. Overall, social support is a prominent theme in literature about breast cancer communication on social media [2].

YouTube is growing as a source of “peer-to-peer health information sharing and support” [3]. The importance of peer support across cancer is widely acknowledged [4]. Breast cancer peer support programs have been shown to be effective in enhancing patients’ quality of life [5], particularly regarding the alleviation of depression and anxiety [6]. Moreover, peer programs are similarly shown to be successful in providing emotional and psychosocial support [7]. Patient support groups can fulfill patient needs and improve quality of life [6,8,9]. Peer education was also found to reduce levels of psychological pain [10]. Research demonstrates that women with breast cancer need and expect online support programs, despite platform-related challenges such as inconsistent content moderation and that “well-organized and tailored” peer support is important to enhance their quality of life [11].

While vlogs about breast cancer may have the potential to serve as peer-to-peer support and provide community, their content, quality, and role in the breast cancer experience are understudied. The objectives of this study were to (1) determine what forms of content about breast cancer tend to be discussed in vlogs, (2) inquire into the reasons why women choose to vlog their breast cancer experiences, and (3) consider the potential for breast cancer vlogs to serve as an alternative or complement to peer-to-peer support, as well as a site of digital community overall.

## Methods

### Study Design

YouTube was searched in incognito mode in November 2023, using the search terms “breast cancer vlog.” Breast cancer vlog creators were identified, and their video characteristics were collected. If the creator produced less than 10 videos, all were included. If they produced more than 10, a maximum of 10 were included, based on viewership and most recent date created. Creators were identified based on whether they produced English-language breast cancer-related videos in the last 5 years. Prior to evaluating the videos in this dataset, reviewers were trained to collect vlog data using a standardized data collection tool. A sample was collected, and data collection quality, as well as procedures for any disagreement in evaluations, were assessed before initiating the formal data collection.

Reviewers collected creator information (ie, age, location, profession, and cancer stage). They collected the video titles; length; date; number of views, likes, and comments; and hashtags; and noted whether the videos were part of a playlist. Reviewers then assessed videos based on consumer details (ie, sponsorships, product recommendations, and endorsements), and the creators’ explanations and discussions of their breast cancer. Engagement levels were considered based on vlog creators’ discussion with their audience, whether they commented on their experience with their audience, or asked the audience to share insights of their own. Types of content (diagnosis, surgery, or other event) were also collected.

Video themes were extracted both deductively and inductively. The themes collected deductively are described in Table 1. Descriptive and content analyses were performed to assess and analyze video content and potential areas where peer-to-peer support may be provided. Reviewers were encouraged to add subthemes inductively in cases where the content exceeded or was more specific than the deductive themes [12]. An additional set of questions was applied to videos that contained advice, in order to characterize the nature, source, and potential validity of the advice provided (Table 2).

**Table 1.** Deductive themes.

Theme	Definition
Appearance	Refers to one’s experiences with their appearance, including how it may have changed over the course of treatment
Mental health	Refers to questions of mental health, including stress, anxiety, depression, or other related issues
Fear of recurrence	Refers to fears or anxieties related to the future possibility of a cancer recurrence
Gender identity	Refers to one’s gender identity, particularly in the context of breast surgery or reconstruction choices
Sexuality	Refers to experiences of sexual intimacy, including how these may have changed over the course of treatment
Fertility	Refers to one’s experiences with fertility or infertility, including fertility treatment
Motherhood	Refers to the specific relationship between mother and child, how to disclose to one’s children, as well as wanting to be a mother
Spousal relationship	Refers to the patient’s relationship with their spouse, including stress on the spouse who takes on a caregiving role
Family relationship	Refers to family-based experiences, such as how one may disclose their diagnosis to their family and how a diagnosis shifts the family dynamic
Path to diagnosis	Refers to the story or experience of being diagnosed with breast cancer, such as discovering a breast lump
Treatment	Refers to a wide array of topics, ranging from treatment choices to the experience of receiving treatment
Adverse effects	Refers to specific experiences with adverse effects, whether due to surgery, chemotherapy, or other forms of treatment

**Table 2.** Advice assessment.

Characteristic	Definition
Vlog creator verbalizes a reference or source for advice.	Refers to the vlog creator verbally explaining where or from whom they learned about the advice provided.
Vlog creator confirms having tried the advice personally.	Refers to the vlog creator describing their own personal experience by following the advice provided.
Proposed advice recommends adding something (addition) or not doing something (omission).	Refers to whether the vlog creator suggests making some sort of addition to their care or cancer management or ceasing or removing an aspect of their care or cancer management.
Proposed advice involves a product to be applied or consumed.	Refers to whether the proposed advice involves applying or consuming a product.
Proposed advice suggests a modification or reduction in the treatment plan.	Refers to whether the proposed advice suggests some sort of modification to or reduction in one’s cancer care management or treatment plan.
Proposed advice is potentially beneficial, neutral, or potentially harmful.	Refers to whether the advice provided is of potential benefit to a patient, neutral, or of potential harm to a patient.

### Ethical Considerations

Institutional ethics review was not required for the completion of the study, as all the data including patient- and disease-specific information and opinions or experiences were volunteered into the public domain by the creators. No patient- or disease-specific information was collected. No identifiable information is published.

## Results

A total of 90 vlogs by 13 vlog creators were included in the study, all of which originated from personal YouTube accounts. The mean (SD) video length was 21.4 (9.1) minutes. The mean (SD) number of views was 266,780 (534,465). The mean (SD) number of comments was 1485 (3422). Hashtags—words or phrases preceded by the “#” symbol that serve to categorize social media content—were included in 35 videos (39%), the majority of which were breast cancer-related. Paid sponsorships were present in 11 videos (12%). Creators promoted their own channel in a large majority of videos (80/90; 89%); for instance, by encouraging channel subscriptions. Most creators (88/90; 98%) included a title that effectively summarized the video topic, meaning that the title described the events discussed in the video. Where mentioned, the range of 20‐29 years was the most common age group (14/27; 52%), followed by 30‐39 years (10/27; 37%). Where mentioned, the most common creator location was the United States of America (25/40; 62%).

Visuals were present in 60/90 videos (67%); of these 60 videos, 34 (57%) included images or videos of vlog creators undergoing treatment (such as receiving chemotherapy or radiation, or undergoing magnetic resonance imaging) as well as physical features of treatment, including port scars, surgical drains, and breast contour after expander placement. A portion of videos (36/90; 40%) included inserted recorded clips, for instance, playing a recording of a phone call with their pathologist discussing results or footage of entering the radiation machine.

Videos were mainly filmed at home (87/90; 97%), at the hospital (28/90; 31%), or in the car (19/90; 21%). It is possible that vlogs were filmed in more than one setting. When filmed at home, vlogs were most often set in the creator’s living room (43/90; 44%), bedroom (32/90; 33%), or kitchen or office (both 20/90; 21%).

In half of the vlogs (45/90, 50%), the creator commented on how their audience makes them feel, and in 44/90 (49%), the creator explained why they decided to make vlogs about their breast cancer experience, the most frequent reasons being: (1) enjoying filming vlogs, (2) wanting to build a community, (3) having a predominantly female viewership, (4) wanting others with cancer to feel less alone, (5) sharing information on surgery, and (6) providing details about signs of their recurrence. For example, one vlog creator described the process of filming and posting vlogs about her metastatic breast cancer as therapeutic “because it feels like she sat and talked to someone about everything on her mind”; this creator also referred to the viewers who have expressed that her vlogs helped them through their own experiences as making her feel like her vlogs have a purpose. It is important to her that her audience—specifically, others facing a similar diagnosis—knows that they are “not alone in this at all.” In 42/90 videos (47%), creators expressed emotion in an explicit way; for instance, one vlog creator filmed her last chemotherapy treatment and was emotional while ringing the celebratory bell and thanking her nurses.

Advice was offered in 52/90 (58%) videos, with the most common topics being cold capping, hair regrowth, clothing, nutrition, mental health habits, chemotherapy preparation, saline soaking for radiation burns, wig use, and cancer prevention. References or sources were rarely cited, with advice usually originating from the video creator themselves. In most cases, the creator confirmed the advice was based on their personal experience. Advice overwhelmingly involved making some sort of addition to one’s care or cancer management rather than an omission. None of the proposed advice was considered of potential harm to patients. Additional details on videos containing advice are outlined in Table 3.

**Table 3.** Advice provided in vlogs.

Characteristic	Frequency (%)	Example
Vlog creator verbalizes a reference or source for advice.	Yes: 3 (5%) No: 49 (95%)	Friend, website, or book
Vlog creator confirms having tried the advice personally.	Yes: 42 (81%) No: 10 (19%)	N/A^a^
Proposed advice recommends adding something (addition) or not doing something (omission).	Yes: 51 (98%) No: 1 (2%)	Cold capping, purchasing products, stretching, attending therapy sessions, using sleep aids, maintaining a positive outlook, or praying
Proposed advice involves a product to be applied or consumed.	Yes: 15 (20%) No: 37 (80%)	Vaseline, compression socks, ginger supplement, cold capping equipment, hair or beauty products, blankets, or pajamas
Proposed advice suggests a modification or reduction in the treatment plan.	Yes: 14 (27%) No: 38 (73%)	Modifying chemotherapy administration method by using a port-a-cath instead of a peripheral venous infusion
Proposed advice is potentially beneficial, neutral, or potentially harmful.	Harmful: 0% Neutral: 24 (46%) Potentially beneficial: 28 (54%)	Reduced risk of deep vein thrombosis, reduced hair loss, and reduced diarrhea

a N/A: not applicable.

Where the cancer stage was mentioned, stage IV was most common (13/90; 14%). Of the 90 videos, 22 (24%) were about a new diagnosis. Chemotherapy was the predominant treatment form discussed in the majority of videos (63/90, 70%); surgery in 58% (52/90), primarily mastectomy (20/52, 38%); and radiation in 30% (27/90). The general adverse effects were discussed in 71% of the videos (64/90). In over half of the videos (50/90; 57%), creators provided a structured definition to some aspect of their treatment.

The most common themes were treatment (77/90; 86%), mental health (73/90; 81%), adverse effects (65/90; 72%), appearance (57/90; 63%), and family relationships (33/90; 37%). Subthemes included young age, finances, the importance of online community support, social life, fear of surgery, egg retrieval, and confidence and redefining beauty standards.

## Discussion

### Principal Findings and Comparison With Previous Works

This study demonstrates that vlogs by women with breast cancer receive significant levels of engagement and represent an important site of online community for women. However, the nature of their content, their authorship, and the potential to be integrated into care plans are underexplored. Our study shows that most commonly discussed themes in breast cancer vlogs include treatment, mental health, adverse effects, and appearance, but that a wide range of subthemes are also present. Moreover, they are often filmed in a personal home setting. These patient-created videos, which included less paid sponsorship than what researchers have identified in previous analyses [12], included detailed and overt expressions about why creating vlogs about their breast cancer is a valuable experience. The production behind vlogs, combined with the settings in which they are filmed, contribute to the feeling of connection between the creator and the audience. That vlogs are often filmed at home may be important to building a sense of community. The seemingly close and casual environment of filming in one’s own living room or bedroom creates a sense of proximity to the vlog creator that is more aligned with the support group model that might not necessarily be present in a professional setting.

There is a rich community-centered aspect to breast cancer vlogs, which may position them as complementary forms of peer-to-peer social support and as unique methods for effective coping. Tailoring peer support to the moments when patients are most in need is crucial [13]. In their study on factors of engagement and patient-reported outcomes in a stage IV breast cancer Facebook group, Kashian and Jacobson [14] conclude, “Optimal social support plays a critical role facilitating engagement in online breast cancer support groups. It is not enough that members exchange social support in online support groups, rather members must exchange the type of support that facilitates effective coping.” Power and Hegarty [15] also found that support programs need to be tailored to the needs of women with breast cancer and have identified “the need to allow more informal sharing to occur in facilitated peer support programs.” Vlogs are easily findable and watchable. While different peer support models and approaches will have varying outcomes on different patients, web-based support without training and/or moderation should be used with caution [16]. YouTube is heavily engaged with for public health reasons. However, there is a need for higher-quality content [17]. Research evaluating YouTube videos about radiotherapy in breast cancer concludes that while videos were inconsistent in following best practice guidelines, YouTube still has potential toward disseminating health information [18]. Research on YouTube videos about radiotherapy in lung cancer draw similar conclusions [19].

It is possible that informal sharing also happens in online spaces, where patients might feel they have more control over how much they can share and can do so in the format, style, narrative, and medium of their choosing. Ziegler and colleagues [20] have shown a moderate positive association between peer support and psychological improvement in cancer patients. People who post and share about their experiences in online communities are not the only ones who benefit from the peer support in these spaces; research shows that “lurkers,” or those who watch or consume content without sharing their own, can also benefit from the advice and insights shared [21]. Many messages in online support groups are requests for information and opinion from those in the same situation and are designed to reach like-minded people [22]. These networks are informal, grow organically, and are accessed when needed.

The use of visuals and additional recorded clips in vlogs, such as the inserted phone recordings or hospital footage, may contribute to a sense of closeness between the creator and their audience. Such instances of additional video editing are indications of vlog creators’ platform expertise and demonstrate the production efforts behind vlog creation [23]. However, in the context of documenting one’s breast cancer experiences, additional video editing may actually serve to provide even more detail for one’s audience: playing a phone recording or showing hospital footage (of, for instance, receiving chemotherapy infusions) allows the audience to see and hear parts of the creator’s breast cancer experience that they were not present for, thereby creating a further sense of closeness with the creator. Inviting one’s audience into private hospital or clinical encounters also holds implications for standardized practices of medical confidentiality. For these creators, there is something important about sharing what tends to be considered private. The perceived closeness afforded by hospital footage and recorded phone calls, for example, may also serve to demystify these experiences for audience members who might be about to begin their own breast cancer treatment. In this context, typical conceptions of what is considered public or private are blurred, and viewers may begin to develop emotional bonds with vloggers they have never met: a phenomenon not unique to vlogs about breast cancer, but which occurs across different types of social media content and platforms [24]. Scholars have discussed the notion of the microcelebrity, particularly the ways in which celebrity and fame are connected to different media [25]. Given this context, it is plausible that breast cancer vloggers may achieve certain levels of microcelebrity status on YouTube, which may result in a feeling of parasocial closeness—defined as “nonreciprocal socio-emotional connections with media figures such as celebrities or influencers”—on the part of the audience, and which can continue in cases where the video creator dies [26].

Key features of breast cancer vlogs, which include explanations of the diagnosis experience, are associated with receiving empathic support from audiences [27]. The emotionally intense context afforded by the medium of video is demonstrated to lead to community-building and social support among vloggers and their audiences [28]. In their study of breast cancer narratives on social media, Ma and colleagues [29] found, “Stories that were longer, less emotionally intensive, told from the cancer survivor’s perspective, with gender identity–related information, describing the act of providing social support, explicitly requesting engagement and/or donation, and using more vivid forms of visuals such as linked images tended to be more engaging.” However, while Ma and colleagues [29] found that emotionally intensive stories may be less engaging, social media research suggests, “Crying and anxiety blogs can function as a means of demonstrating vloggers’ ‘authenticity,’ and thus fostering valuable intimacy between vloggers and their audience” [30]. The sense of intimacy shared by vloggers—especially when demonstrating explicit emotion in their vlogs—is valuable in this context because it serves to develop a bond between the creator and the audience, who may decide to begin regularly following the creator’s breast cancer trajectory. In our study, creators explicitly expressed emotion in almost half of the videos. Emotion is an important part of the breast cancer stories that vlog creators tell, and it resonates with their audiences: several videos in this dataset, particularly those relating to metastatic disease and end of life, were challenging for reviewers to watch and remained distinctly memorable after data collection was completed. The vulnerability and emotion present in these vlogs—and thus, their relatability—may provide a form of connection between creator and audience that health care professionals cannot provide due to the objectivity they have to maintain in delivering care.

Vlogging about breast cancer may also hold therapeutic value [31]. Creators in this dataset have discussed what they perceive to be the benefits of vlogging, among which is the idea that vlogging is a way to speak about everything on their minds, not unlike sitting and chatting with someone. When creators hear from their audience that watching their vlogs helped them to feel less alone, or helped them in their own journeys, they express that this audience response helps them to feel like their vlogs have a purpose. Vlogs are a source of support and can help patients cope with isolation not only in dealing with cancer, but also with other chronic illnesses such as fibromyalgia, diabetes, and HIV [32-34]. The value of vlogging about breast cancer can extend beyond patient communities to health care settings; one reviewer, a surgical resident, commented that watching the vlogs in this dataset was important to their training experience. For this reviewer, seeing how patients understand the course of their treatment as explained to them by their doctor/surgeon, what they focus on, what they fear, and how they take the information they have received and share it in a digestible way with their viewers was very useful, and would contribute to how they engage with cancer patients going forward. In this way, watching vlogs may also hold value in medical training settings, as examples of voluntarily provided patient experience.

Questions of how to handle the transfer of information in vlogs were considered throughout this study’s design. In some cases, reviewers were able to anecdotally or casually identify some vloggers who provided high-quality explanations of their cancer and its treatment, to the point that the reviewer would suggest it to their patients if needed. That being said, it is challenging even for health care professionals to evaluate the quality of information as it is presented intertwined with personal experiences (often indistinguishable) in experience-based videos. Validated tools for evaluating YouTube video quality now exist; however, Gabarron and colleagues [35] demonstrated, in their 2013 review, that as recently as 10 years ago, guidelines for such evaluations were “unclear and not standardized.” While standardized instruments such as the DISCERN tool and the Patient Education Materials Assessment Tool can be used to evaluate different forms of media, their applicability is limited in regards to patient-created, experiential content. Vlogs are an experience-based media format grounded in personal storytelling, where it is often difficult to distinguish information from advice or lived experience. As such, the goals behind both creating vlogs and watching them may be more closely related primarily to building and seeking forms of community and connection, rather than imparting information or learning about medical facts. Nevertheless, our assessment revealed that the majority of advice offered in vlogs consisted of patient-centered concerns and personal preferences that were unlikely to affect cancer treatment trajectories. The proposed advice was not found to be of potential harm to patients (even if questionably beneficial) and was largely experiential.

### Limitations

The limitations of this study include restrictions based on language and challenges related to collecting demographic data. This study was limited to English-speaking videos, which may influence views or perceptions of the breast cancer experience. In addition, because vloggers may or may not disclose specific information such as their age, nationality, location, profession, or cancer stage, the consistent collection of these data points was not possible. Future research on breast cancer vlogging practices in specific linguistic, geographic, or ethno-cultural communities represents an opportunity to understand the circulation of health information within communities that may not have easy access to mainstream health services. While some of the peer support literature cited in this paper predates the development and widespread use of social media, the authors contend that this earlier foundational literature functions as a precursor to contemporary understandings of the benefits of social media communities, their effects on patients’ outlooks, as well as their reasons for participating in them.

Given that the purpose of vlogging is not to educate one’s peers but to share experiences and build an online community, the potential for using currently existing standardized tools to assess information quality is limited. In light of the growth of online peer-support communities and the lack of methodology regarding the quality and accuracy of information that is woven into patient experiences reported in the vlog format, there is a need for the development of a methodology that specifically validates the quality of information transferred in these settings. The next phase of our research will address breast cancer vlogs, and other cancer-related media content, created by and from the perspectives of health care professionals where, in contrast, health care professionals have a primary goal of transferring information to support patients. Future research may evaluate the differences in engagement metrics (such as views, likes, or comments) between breast cancer video content created by patients for patients and content created by health care professionals.

### Conclusion

This study demonstrated that the awareness of and dedication to building community that vlog creators show in this context, as well as the personal nature of their storytelling, their advice and suggestions, and their discussions of wide-ranging yet specific topics all position vlogs by women with breast cancer as a potential resource for peer-to-peer support in breast cancer. The experiences of both creating and watching breast cancer vlogs hold significant potential benefits for peer-to-peer support in breast cancer care. This study aligns with Kashian and Jacobson’s [14] conclusions, which suggest that given the association between optimal social support and community engagement, “Hopefully practitioners will use this information to encourage patients to join quality online support groups for positive experiences.” While there are risks associated with consuming online content, the potential benefits of community and support offered by breast cancer vlogs should not be overlooked. Future research will consider patient perspectives and further address how the specific themes discussed in vlogs may be used to improve the cancer care experience for breast cancer and other cancers.
